# C-reactive protein during the first 6 postoperative days after total hip arthroplasty cannot predict early periprosthetic infection

**DOI:** 10.1007/s00402-022-04565-4

**Published:** 2022-08-09

**Authors:** Sebastian Rohe, Sabrina Böhle, Georg Matziolis, Benjamin Jacob, Georgi Wassilew, Steffen Brodt

**Affiliations:** 1 Orthopaedic Department of the Waldkliniken Eisenberg, University Hospital Jena, Campus Waldkliniken Eisenberg, Klosterlausnitzer Straße 81, 07607 Eisenberg, Germany; 2 Department of Orthopaedic Surgery, University Hospital Greifswald, 17475 Greifswald, Germany

**Keywords:** Total hip arthroplasty, C-reactive protein, Periprosthetic infection, CRP, Acute postoperative PJI, Revision surgery

## Abstract

**Introduction:**

Periprosthetic joint infection (PJI) after total hip arthroplasty (THA) remains a serious complication in orthopaedic surgery. C-reactive protein (CRP) is widely used as a marker to screen for inflammatory complications. The early postoperative course is well known, but knowledge about the predictive value of CRP in the first 6 postoperative days for detecting an acute postoperative PJI is lacking.

**Methods:**

We retrospectively analyzed the inpatient course of CRP of all primary THA and THA with acute PJI within 28 days in our hospital from 2013 to 2021. A receiver-operating curve (ROC) analysis was performed and the best CRP threshold for detecting an acute PJI based on Youden’s-index was calculated and an area-under-the curve (AUC) analysis of the threshold was performed.

**Results:**

33 of 7042 patients included had an acute PJI within 28 days. Patients with acute PJI were older, had a higher BMI and longer operation time and suffered more often from diabetes mellitus. A preoperatively elevated CRP was a risk factor for PJI. CRP was significantly higher in the PJI group on postoperative days 3 and 5. Threshold values were calculated to be 152 mg/l on day 3 and 73 mg/l on day 5. However, these values had a low sensitivity (75%, 76%) and specificity (67%, 61%).

**Conclusion:**

Especially considering the decreasing length of stay after THA, the question of the usefulness of regular inpatient CRP checks arises. AUC analysis of the ROC showed a poor diagnostic accuracy in almost all cases. Only the dynamic analysis of the maximum CRP value to the lowest CRP value with a decrease of 102.7 mg/l showed a fair accuracy. This calls into question the clinical relevance of CRP in the first postoperative week for detection of acute postoperative PJI.

## Introduction

Periprosthetic joint infection (PJI) after total hip arthroplasty (THA) remains a serious complication in orthopaedic surgery. C-reactive protein (CRP) is widely used as an inflammatory marker to screen for inflammatory complications [1–3]. Postoperatively, CRP levels are influenced by surgical trauma and the associated inflammatory tissue response, making the diagnosis of acute periprosthetic joint infection difficult [4, 5]. After THA, it is well known that there is initially an increase in CRP levels until the 3rd postoperative day, followed by normalisation by the 3rd week [4–6]. Furthermore, this increase has been shown to be sex dependent [7]. C-reactive protein (CRP), an acute phase protein, is predominantly produced by hepatocytes and triggered by interleukin-6 (IL-6). It has a very low blood plasma concentration (0.8 mg/L to 3.0 mg/L) in healthy adults and is rapidly elevated in case of acute inflammation, infection, malignancy and traumatic injury or surgery with high interindividual variations. Moreover CRP has a long half-life and stable levels with negligible circadian variation [8]. CRP exerts its effect by marking bacteria for opsonization, complement activation and binding to damaged cell membranes [9]. The rapid increase in synthesis within hours of tissue injury or infection suggests that it contributes to host defense, and that it is part of the innate immune response [9, 10]. Thus, raised CRP levels are considered to be a useful parameter in detecting complications of bacterial infection after surgery and to reflect the extent of surgical trauma [9, 10]. If the value remains high or if there is a new rise on days 7 or 8, systemic infection should be suspected [11]. Clinical signs, such as erythema, drainage, and pain, also do not provide a reliable indicator of the presence of a postoperative infection, especially in the early postoperative period [12].

In 2018, the Second International Consensus Meeting (ICM) of the Musculoskeletal Infection Society (MSIS) developed new criteria for the diagnosis of PJI, which have since been investigated in various studies with regard to their validity [12, 13]. A threshold value of 10 mg/l was set for a chronic PJI and 100 mg/l for an acute PJI as one of seven criteria. However, the threshold for the diagnosis of acute PJI is only a recommendation [12, 13].

For acute PJI, Yi et al. calculated a cut-off of 93 mg/l within the first 6 weeks with a specificity of 100% and sensitivity of 88% and Sukhonthamarn et al. calculated within the first 90, 45 and 30 days 39.8, 44.9 and 50.7 mg/l as cut-offs with a specificity of 95%, 89% and 83% and sensitivity of 91%, 86% and 85%, respectively [14, 15]. Chapman et al. developed a formula to calculate a cut-off for an infectious CRP course, considering the initial dynamics of CRP progression in treated femoral neck fractures: CRP cut-off = 500 / (postoperative day) [16]. None of the studies dealing with THA take into consideration the initial postoperative period. However, this is of immense importance, especially regarding fast-track surgery and the evaluation of the benefit of postoperative CRP control for the detection of acute postoperative PJI within 28 days after index surgery. Within these days a potential debridement, antibiotics and implant retention procedure (DAIR) can be recommended as implant retaining therapy [17–20]. However, there is still a lack of valid CRP thresholds in this initial postoperative period after index surgery for diagnosis of an acute PJI.

Therefore, the following study deals with the diagnostic value of CRP in the first 6 postoperative days during the inpatient stay for diagnosing acute postoperative PJI and with the risk stratification resulting from a preoperatively increased CRP for the occurrence of acute postoperative PJI.

## Materials and methods

**Fig. 1:**
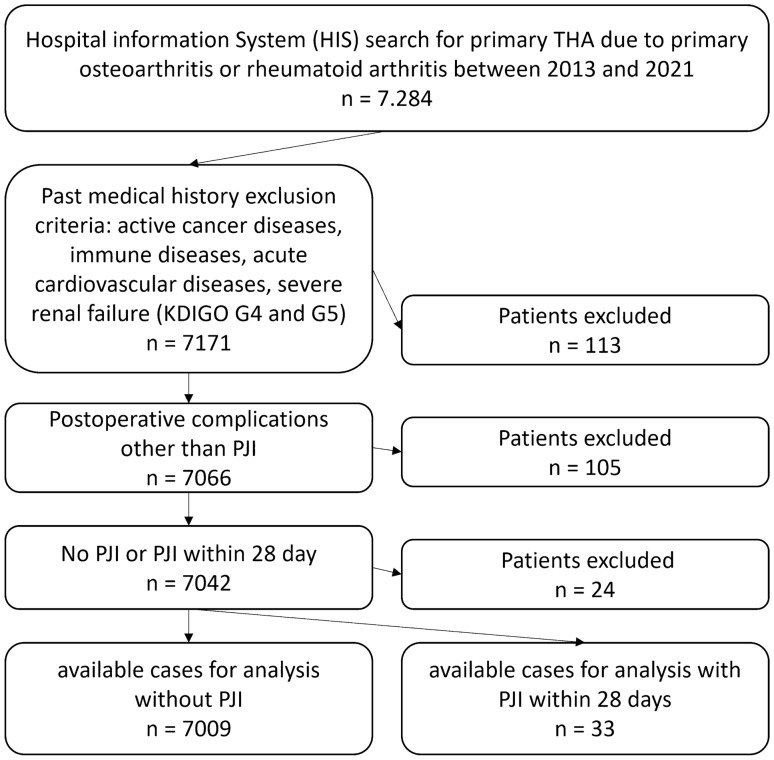
Flowchart of data recruitment

In this retrospective case–control study, all patients who underwent a primary THA because of primary osteoarthritis and secondary osteoarthritis due to rheumatoid arthritis in a maximum care orthopaedic hospital between 2013 and 2021 were initially included after informed consent and approval by the local ethics committee (Reg. No. 2021-2310). The following data were collected from the hospital information system: age, gender, height, weight, BMI, HbA1c, preoperative glomerular filtration rate (GFR), operation time, past medical history (rheumatoid arthritis, osteoporosis, diabetes mellitus, active cancer diseases, immune diseases, acute cardiovascular and lung diseases, severe renal failure), CRP values, peri- and postoperative complications and revision surgery. Periprosthetic infections within 28 days leading to revision were considered as acute postoperative PJI according to the ICM criteria 2018 [13], as a DAIR procedure can still be performed during this interval [17–20]. Renal failure was classified according to KDIGO criteria [21]. Diabetes mellitus was diagnosed by positive past medical history or HbA1c values greater than 7.5% preoperatively [22]. Obesity was classified according to WHO recommendations [23]. Patients with peri- and postoperative complications other than PJI (dislocations, fractures, falls, severe postoperative renal failure (KDIGO G4 and G5), pneumonia, and urinary tract infection), active cancer diseases, immune diseases (except rheumatoid arthritis), acute cardiovascular diseases and severe renal failure (KDIGO G4 and G5) were excluded to avoid bias in CRP values (Fig. 1). To obtain a threshold value that is easy to apply clinically, all other patients were included in the final analysis regardless of their secondary diseases.

Blood samples were taken in routine care preoperatively and on postoperative days 1, 3, 6 and after shortening the standard length of stay on day 5 or 4 depending on discharge. The CRP value was measured in the hospital’s own laboratory using a qualitative visual latex agglutination test. A pathological CRP was defined as higher than 5 mg/l (0.5 mg/dl). The effective measuring range of the method used is estimated to be 0.3 mg/l to 350 mg/l. The lowest limit of detection was 0.2 mg/l, and the lowest identification threshold was 0.3 mg/l. The effective measuring range and identification threshold were determined according to the explicit requirements of the medical device directive EP17-A of the Clinical and Laboratory Standards Institute.

### Statistical analysis

Data management was done using Microsoft Excel 365 and statistical analysis using IBM SPSS 27. Chi^2^ tests were used for unpaired nominal variables and the Wilcoxon rank sum test was used for metric and ordinal variables to determine the level of significance. Logistic regression analysis was performed for potential confounders. A value of *p* < 0.05 was defined as significant. To determine the diagnostic value (sensitivity and specificity) of detecting acute PJI within 28 days, receiver-operating characteristics (ROC) curves were calculated for CRP values from preoperative to 6th postoperative day, the difference between postoperative CRP maximum and minimum, and the difference between preoperative and highest postoperative CRP values. Missing values were automatically excluded before analysis via SPSS. Subsequently, the optimal CRP cut-off values with the highest sensitivity and specificity were determined as a measure of test performance using Youden’s index [24] and the AUC was calculated. Test accuracy was rated as follows: AUC between 0.90 and 1.00 = excellent discrimination ability, and AUC from 0.80 to 0.90, 0.70 to 0.80, 0.60 to 0.70 and 0.50 to 0.60 = good, fair, poor and fail discrimination ability, respectively [25]. Finally, potential thresholds for screening purposes with a sensitivity of 90%, which is in the authors opinion a minimum of accuracy, were determined from the initially calculated Youden’s indices of the measured CRP level and the area-under-the-curve (AUC) analysis of the thresholds was performed.

## Results

**Fig. 2:**
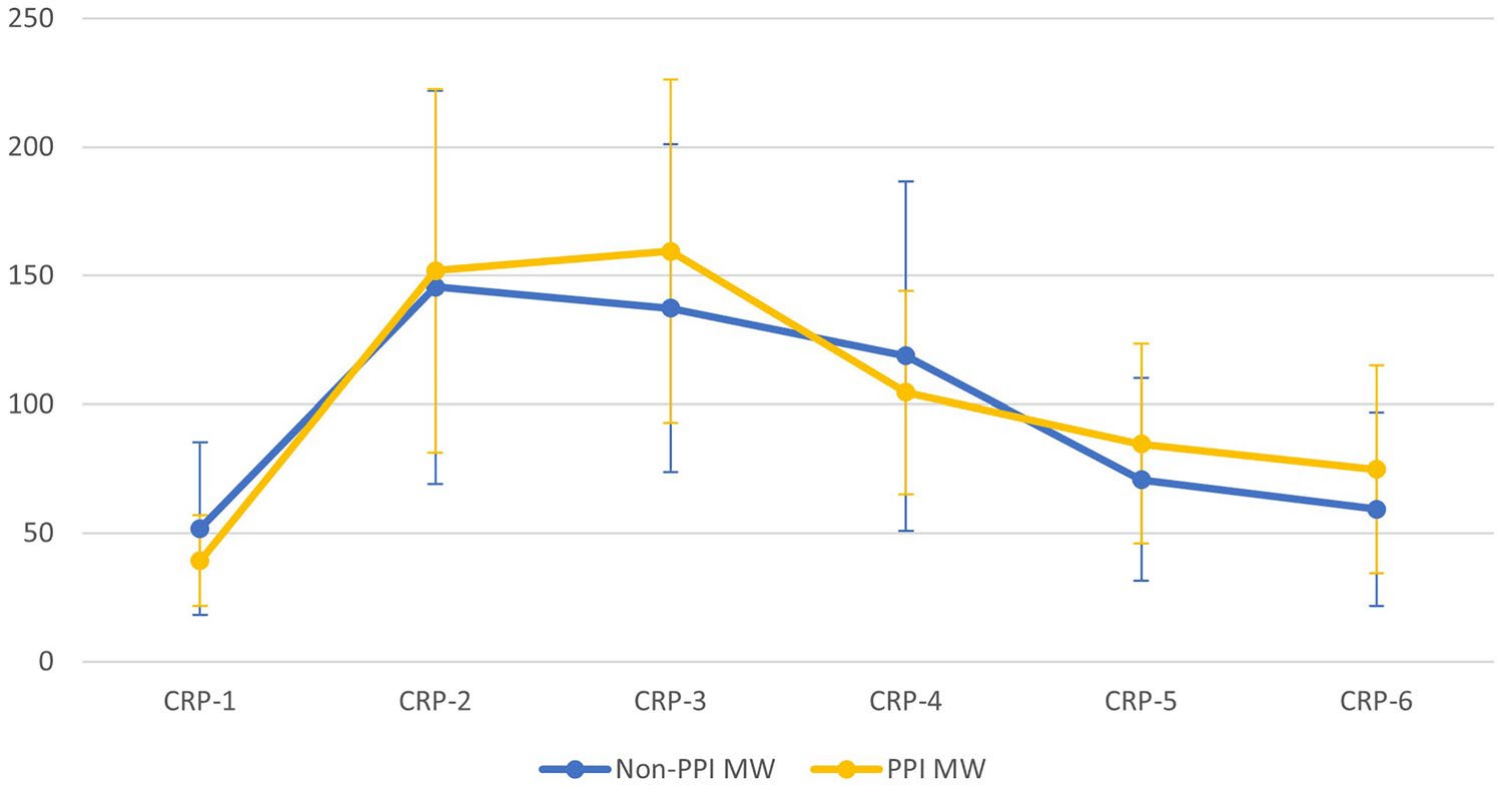
Postoperative course of C-reactive protein (CRP [mg/l]; MW: mean value; PPI: periprosthetic joint infection)

**Fig. 3:**
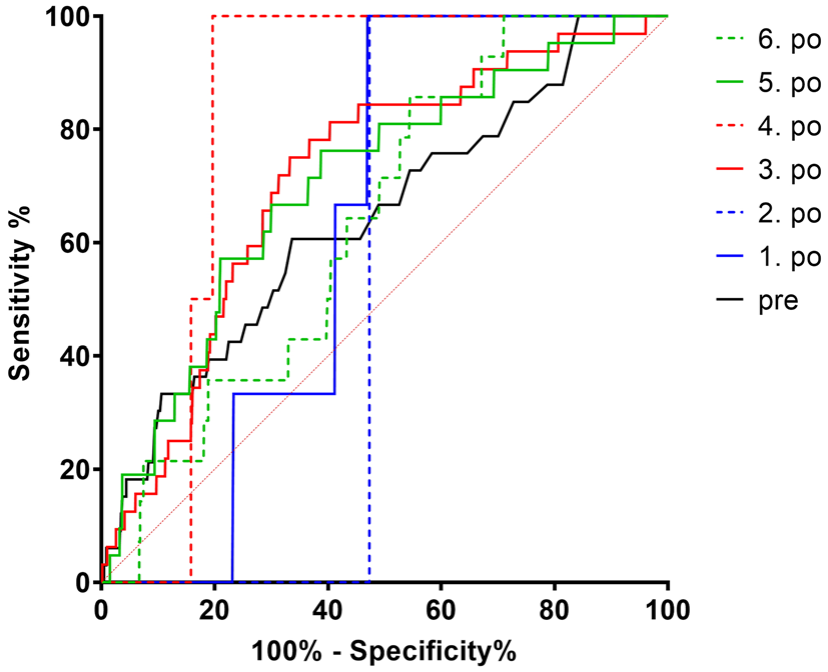
Germ-dependent postoperative CRP course; MW: mean value; PPI: periprosthetic joint infection)

Initially, 7284 patients were included. 242 patients were excluded due to peri- and postoperative complications, past medical history, or a late PJI after more than 28 days, so 7042 patients were considered in the final analysis. 33 patients had an acute PJI with revision surgery within 28 days (Fig. 1). Baseline characteristics of the patients are shown in Table 1. Patients with acute PJI within 28 days were more likely to have diabetes mellitus, morbid obesity (BMI > 35 kg/m^2^), and moderate renal insufficiency, as well as a higher BMI, while patients with a mild obesity (BMI 25 – 30 kg/m^2^) had a lower risk for PJI (Table 1). After logistic regression analysis for confounding factors age (*p* < 0.022), BMI (*p* < 0.001), operation time (*p* < 0.027), and diabetes mellitus (*p* < 0.048) remained significant, while moderate renal failure (*p* < 0.322) lost significancy. The course of postoperative CRP values is shown in Fig. 2. CRP values were significantly elevated in the PJI group preoperatively, as well as on the 3rd and 5th postoperative day (Fig. 2, Table 1). Germs responsible for PJI are reported in Table 2. The germ-dependent CRP course is shown in Fig. 3. A significant difference was reported for streptococcus spp. (*p* < 0.027) and for pseudomonas aeruginosa (*p* < 0.03). However, it was only one detection of pseudomonas aeruginosa outside a mixed germ population.

**Table1:** Baseline characteristics

	Non-PJI	PJI	Significance level	Likelihood ratio
Total	7009	33		
Male	3219 (46%)	14 (42%)	0.896	0.23
Age [y]	65.3 ± 11.4	69.9 ± 9.3	0.018	–
Operation time [min]	59.9 ± 21.9	73.7 ± 30.7	0.002	–
Osteoporosis	399 (5.7%)	4 (12.1%)	0.113	1.94
Diabetes mellitus	900 (12.8%)	11 (33.3)	0.001	9.14
HbA1c [%]	5.9 ± 0.7	6.2 ± 1.0	0.319	–
BMI [kg/m^2^]	28.7 ± 4.9 (min 15.7, max 60.4)	32.7 ± 6.4 (min 21.5, max 49.7)	0.001	–
Obesity (BMI > 25)	5247 (74.9%)	27 (81.8%)	0.358	0.907
BMI 25–30 (Overweight)	2836 (40.4%)	4 (12.1%)	0.001	–12.89
BMI 30–35 (Class I Obesity)	1731 (24.7%)	13 (39.4%)	0.051	3.44
BMI 35–40 (Class II Obesity)	548 (7.8%)	7 (21.2%)	0.004	5.77
BMI > 40 (Class III Obesity)	179 (2.6%)	3 (9.1%)	0.018	3.33
Rheumatoid arthritis	167 (2.4%)	2 (6.1%)	0.168	1.34
Renal failure (KDIGO G3)	266 (3.8%)	4 (12.1%)	0.013	4.01
Pre-operative CRP-value [mg/dl]	3.5 ± 3.9 (min 0.3, max 34.3)	5.9 ± 5.9 (min 0.9, max 26.5)	0.004	–
CRP 1. p.op. day [mg/dl]	56.9 ± 32.6 (min 0.3, max 338.7)	43.3 ± 8.4 (min 34.0, max 50.4)	0.440	–
CRP 2. p.op. day [mg/dl]	133.2 ± 63.6 (min 0.4, max 357.9)	119.0 ± 0.0 (min 119, max 119)	0.925	–
CRP 3. p.op. day [mg/dl]	134.1 ± 63.9 (min 0.3, max 593.6)	181.6 ± 64.7 (min 46.1, max 344.3)	0.001	–
CRP 4. p.op. day [mg/dl]	112.9 ± 67.8 (min 0.3, max 408.2)	164.5 ± 11.5 (min 156.3, max 172.6)	0.115	–
CRP 5. p.op. day [mg/dl]	70.4 ± 39.3 (min 0.3, max 461.6)	96.3 ± 40.9 (min 29.8, max 190.5)	0.001	–
CRP 6. p.op. day [mg/dl]	58.5 ± 36.4 (min 0.4, max 325.4)	69.2 ± 26.7 (min 36.9, max 113.2)	0.077	–

*HbA1c* haemoglobin type A1c for diabetic control, *BMI* Body mass index, *KDIGO* Kidney Disease Improving Global Outcomes Score, *CRP* C-reactive protein

**Table 2:** Germs responsible for PJI

Germ	Number of detections
Staphylococcus epidermidis	11
Staphylococcus aureus	7
Staphylococcus capitis	1
Streptococcus spp.	5
Pseudomonas aeruginosa	3
Proteus mirabilis	4
Enterobacter spp. (Serratia spp., Escherichia spp., Klebsiella spp.)	8
Enterococcus spp.	4
Corynebacterium	1
Nondetectable	3

*spp* subspecies

**Fig. 4:**
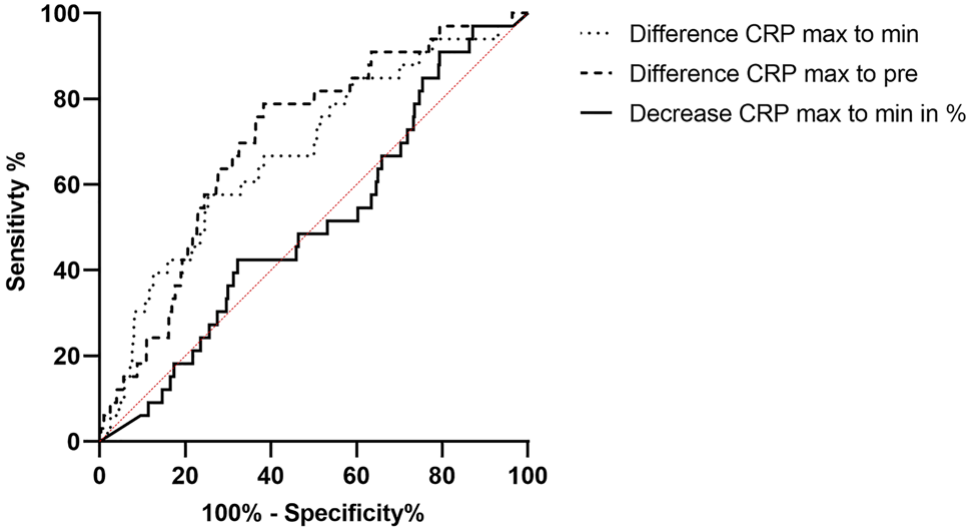
ROC analysis of pre- and postoperative CRP values

The analysis of the ROC curve was performed on 7042 patients after the above-mentioned exclusions and showed only a low diagnostic predictive value for the CRP values on all days with best results on day 3 and 5 and preoperatively. These CRP thresholds, calculated according to Youden, showed only low specificity and sensitivity (Table 3, Fig. 4). After calculating the optimal CRP cut-off an AUC analysis of these CRP cut-offs was performed with only poor diagnostic accuracy or failed accuracy (Table 3). Due to only small amounts of data in the acute PJI group, no relevant results on the accuracy of a CRP cut-off could be calculated on postoperative days 1, 2 and 4. Furthermore, Youden’s index was determined to classify the test quality (–1 to + 1), whereby a value close to 1 indicates a high test quality (Table 3) [24].

**Table 3:** ROC curve analysis and CRP cut-offs via Youden’s index (Fig. 4)

Day	Youden index	CRP cut-off [mg/l]	Sensitivity	Specificity	AUC (95% CI) Sign
Pre	0.27	3.5	61%	66%	0.643 (0.546–0.740) 0.004^a^
1	0.23	33.9	100%	23%	0.371 (0.253–0.489) 0.440^a^
2	0.47	118.7	100%	47%	0.473 (0.434–0.512) 0.925^a^
3	0.42	152.1	75%	67%	0.714 (0.633–0.796) 0.001^a^
4	0.21	67.4	100%	21%	0.823 (0.776–0.869) 0.115^a^
5	0.37	73.2	76%	61%	0.704 (0.595–0.813) 0.001^a^
6	0.31	47.9	86%	45%	0.637 (0.526–0.748) 0.077^a^

^a^classifying value = 0,5 in CI, 0.90 to 1.00 excellent, 0.80 to 0.90 good, 0.70 to 0.80 fair, 0.60 to 0.70 poor and 0.50 to 0.60 fail

*ROC* receiver-operating curve, *CRP* C-reactive protein, *AUC* Area under the Curve, *CI* confidence interval

**Fig. 5:**
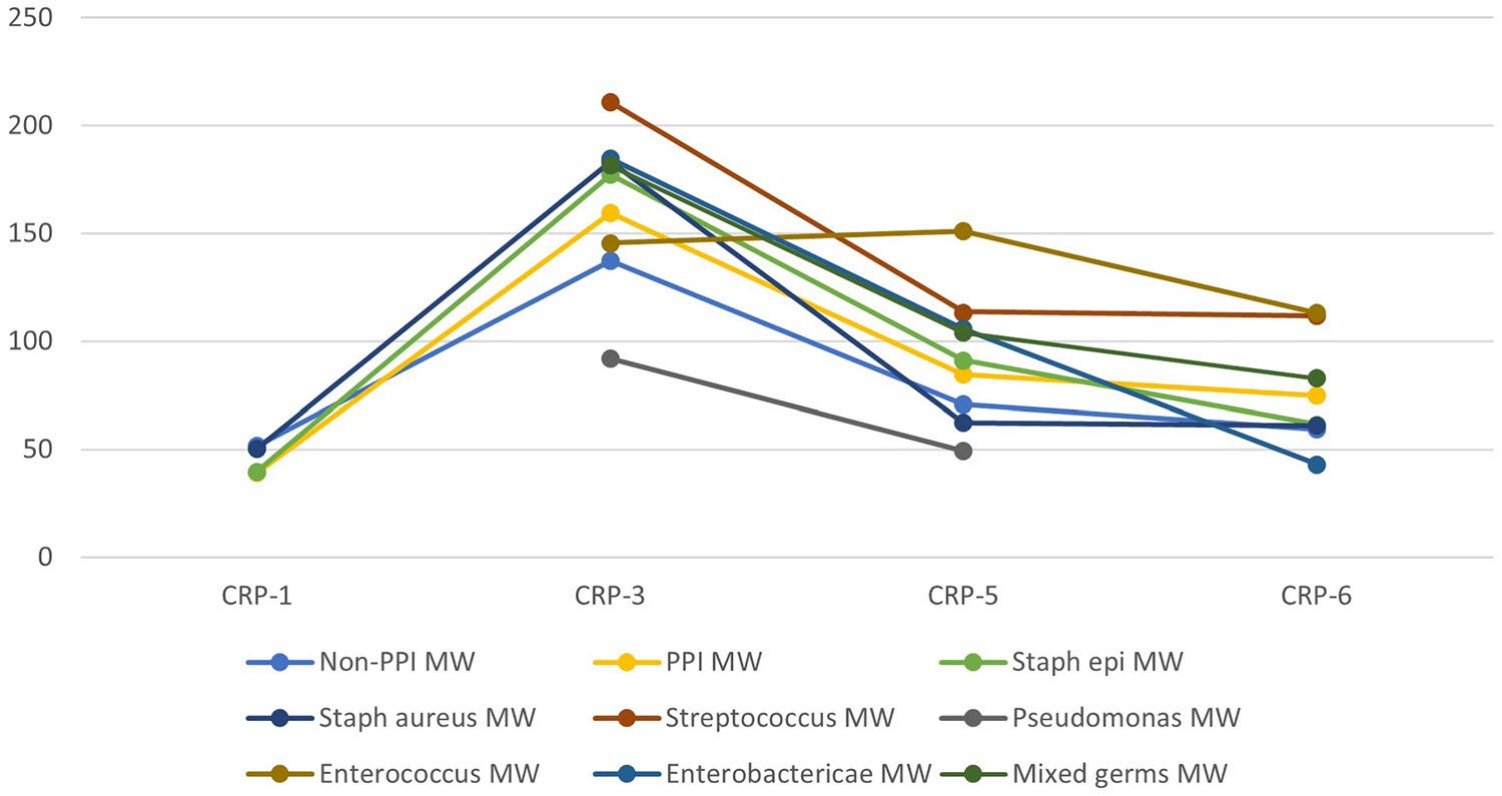
ROC analysis of CRP dynamics

**Fig. 6:**
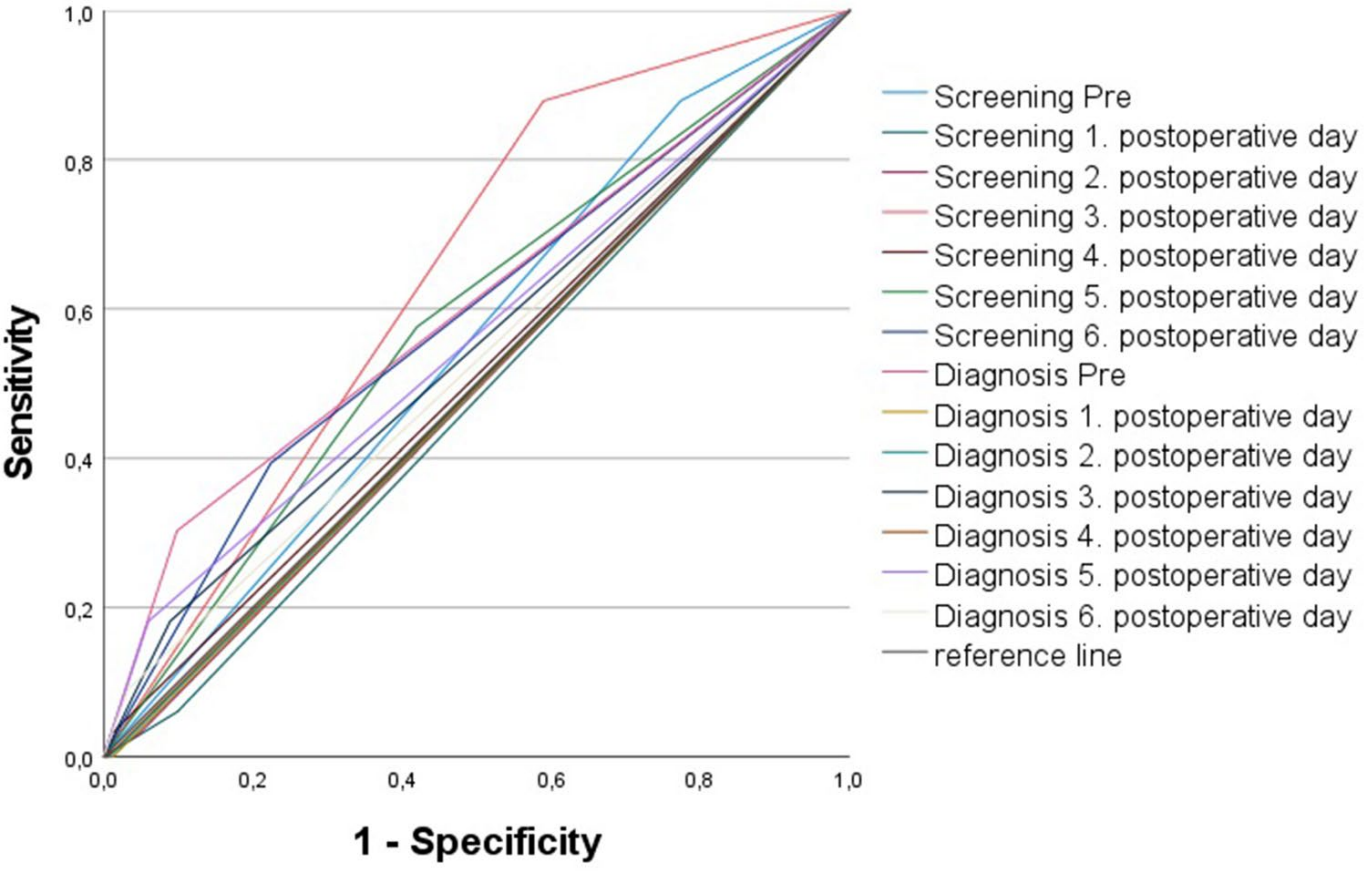
ROC analysis of potential thresholds for screening and diagnostic purposes

Subsequently, an ROC analysis of the dynamics of the CRP courses was performed (difference postoperative CRP maximum to minimum, difference preoperative to postoperative maximum, decrease from maximum to minimum in percent) and showed significant differences (Table 4). However, the clinical relevance was again questionable (Table 4, Fig. 5). In addition, we calculated the potential thresholds for specificity and sensitivity of 90% each, which is in the authors’ opinion the minimum test accuracy needed for a screening or diagnostic test and to visualize the poor corresponding sensitivity and specificity, respectively (Table 5). The AUC analysis in comparison to the ICM MSIS diagnostic criteria from 2018 showed a fail or only a poor diagnostic accuracy (Table 5, Fig. 6).

**Table 4:** ROC curve analysis of the dynamics of the CRP values and their thresholds (Fig. 5)

Dynamic difference	AUC (95% CI) + sign	Threshold	Sensitivity	Specificity
Diff. Pre—Max	0.706 (0.622 to 0.709) 0.001^a^	137.5 mg/l	81%	61%
Diff. Max– Min	0.686 (0.593 to 0.779) 0.001^a^	102.7 mg/l	57%	76%
CRP decrease Max–Min in %	0.471 (0.371 to 0.571) 0.586^a^	41.8%	60%	42%

*ROC* receiver-operating curve, *CRP* C-reactive protein, *AUC* Area under the Curve, *CI* confidence interval; Diff: difference

^a^classifying value = 0.5 in CI, 0.90 to 1.00 excellent, 0.80 to 0.90 good, 0.70 to 0.80 fair, 0.60 to 0.70 poor and 0.50 to 0.60 fail

**Table 5:** CRP cut-offs for 90% sensitivity (screening) and 90% specificity (diagnosis)

90% Specificity				90% Sensitivity		
p.o. day	CRP [mg/l]	Sensitivity	AUC (95% CI) Sign	CRP [mg/l]	Specificity	AUC (95% CI) Sign
Pre	7.5	30%	0.602 (0.494–0.710) 0.042	1.1	21.3%	0.553 (0.462–0.644) 0.296
1	96.4	< 1%	0.494 (0.396–0.591) 0.897	34.1	23.3%	0.481 (0.385–0.576) 0.700
2	220.5*	< 1%*	0.496 (0.398–0.593) 0.929	119.0*	47.3%*	0.492 (0.394–0.589) 0.867
3	224.4	19%	0.546 (0.441–0.651) 0.359	99.7	34.3%	0.644 (0.565–0.724) 0.004
4	204.4*	< 1%*	0.497 (0.399–0.596) 0.958	156.5*	80.4%*	0.510 (0.409–0.611) 0.842
5	116.4	29%	0.561 (0.454–0.667) 0.228	49.3	30.7%	0.578 (0.480–0.675) 0.123
6	100.2	21%	0.529 (0.425–0.632) 0.568	39.4	32.3%	0.585 (0.481–0.688) 0.093

*CRP* C-reactive protein; *p.o* postoperative

^a^classifying value = 0,5 in CI, 0.90 to 1.00 excellent, 0.80 to 0.90 good, 0.70 to 0.80 fair, 0.60 to 0.70 poor and 0.50 to 0.60 fail

^*^Low data volume;

## Discussion

Our study addressed the initial postoperative relevance of CRP and showed that it is not a reliable marker for the detection of acute postoperative PJI and also failed to show a better accuracy than clinical findings [26, 27]. The reason for this inaccuracy is the simultaneous release of CRP due to surgery-related tissue damage as well as infection-related CRP release. Thus, CRP reacts to damaged cell membranes, an activated complement cascade and bacterial surface proteins [9, 10]. Furthermore the release of CRP in the liver is mainly IL-6 dependent and varies highly between individuals [9, 10].

The well-known postoperative CRP course with its maximum on day 3 was confirmed. Furthermore, our calculated optimal cut-off values on day 5 and 6 approached the MSIS recommendation of 100 mg/l [12]. The best correlation with our results was Chapman's formula with cut-off = 500/(postoperative day) on days 3, 5 and 6, despite their reference to treated femoral neck fractures [16]. If we consider the 5th postoperative day according to Chapman et al. with a cut-off value of CRP 100 mg/l, which also corresponds to the MSIS recommendation [12], our ROC curve (Fig. 4) yields a specificity of 84% and a sensitivity of 24%. However, the diagnostic quality in terms of specificity and sensitivity leaves much to be desired for all the thresholds determined in the early postoperative interval. Furthermore, a high specificity is indispensable when deciding on appropriate therapy. Therefore, CRP cut-off and sensitivities were calculated with a specificity of 90%, which could justify further therapies from the authors' point of view (Table 5). These were again of little clinical relevance, due to the very low sensitivity. Also, the analysis with 90% sensitivity as a potential screening test showed no clinically relevant advantages of a postoperative CRP measurement (Table 5).

Previous analysis of Sukhonthamarn, Xu and Yi showed a higher diagnostic accuracy, especially in the ongoing CRP course after 30 days at the earliest [14, 15, 28]. In our subsequent course, the diagnostic quality increased with higher sensitivity and specificity, so that these results seemed reasonable. Sukhonthamarn et al. examined acute PJI within 90 days and determined a CRP threshold for PJI of 39.8 mg/l with a sensitivity of 91% and specificity of 87% which changed only slightly within 45 (44.9 mg/l, Sens.: 82%, Spec.: 89%) and 30 days (50.7 mg/l, Sens.: 82%, Spec.: 90%) [15]. Xu et al. reported a sensitivity of 86.5% at a cut-off of 20 mg/l after 2 to 6 weeks and a lower detection rate when using the MSIS criteria of 100 mg/l with a sensitivity of 55% [28]. In contrast, Yi et al. calculated a cut-off value of 93 mg/l for PJI within the first 6 weeks after arthroplasty with a sensitivity of 88% and a specificity of 100% [14].

In our study, a preoperative cut-off value of 3.45 mg/l was found to be most predictive for acute PJI with a sensitivity of 61% and specificity of 66%. A clinically easy-to-establish cut-off value of 5 mg/l would result in a sensitivity of 39% and specificity of 80%, and 10 mg/l in a sensitivity of 18% and specificity of 95% in our study group. Pfitzner et al. showed in a retrospective matched-cohort analysis that an elevated preoperative CRP value above 5 mg/l is an independent risk factor for periprosthetic infection [29]. This was also confirmed by our results with an increased preoperative CRP value in the acute PJI group (3.5 mg/l vs. 5.9 mg/l). An elevated preoperative CRP value was already reported as risk factor for septic complications in cardiac surgery [30], for early PJI alongside prolonged surgery time and higher BMI after hemiarthroplasty in elderly patients [31], and as a risk factor for surgical site infections after general, oncologic, vascular and orthopaedic trauma procedures [32].

Furthermore, the PJI group showed a higher age and BMI, a longer operating time, a higher prevalence of morbid obesity, diabetes mellitus and moderate renal insufficiency. These factors were significantly associated with the occurrence of an early PJI. Kong and Resende et al. already identified obesity, diabetes mellitus and surgery time as risk factors in two meta-analyses [33, 34]. Concerning obesity, our population showed an increased risk of PJI with a BMI > 35 kg/m^2^ (and a trend with BMI > 30 kg/m^2^) and a decreased risk of PJI with a BMI between 25 and 30 kg/m^2^. This “obesity paradox” was recently published for THA and TKA [35] and confirms our results. Rheumatoid arthritis, male sex and younger age were also mentioned as risk factors, but these were not confirmed in our study population [33, 34]. Cavanaugh et al. showed that renal function has an influence on the occurrence of PJI, as occurred in our study population, but this lost significance after logistic regression analysis for confounding factors [36].

Limitations of our study are the retrospective design, as well as the omission of detailed confounder considerations for the purpose of a general clinically easy-to-use cut-off value. Other limitations are the small data sets of postoperative CRP on days 1, 2 and 4 and the small number of cases in the PJI group. There is also a possible bias due to PJI treatment in other hospitals.

## Conclusion

Especially regarding the decreasing length of stay after THA, the question of the usefulness of regular inpatient CRP checks arises, since the significance regarding an acute PJI was shown to be of minor importance. AUC analysis of the ROC showed in almost all postoperative cases only a poor diagnostic accuracy. Only the dynamic analysis of the maximum CRP value to the lowest CRP value with a decrease of 102.7 mg/l showed a poor accuracy. This laces in doubt the clinical relevance of postoperative CRP for detection of PJI in the first postoperative week. For preoperative CRP evaluation, a threshold of 10 mg/l showed a sensitivity of 18% and specificity of 95% in our study population and should be taken into consideration before surgery.

## Abbreviation

LOE: IIIc retrospective diagnostic case–control study
